# Supplementary material for: Genetic Restoration of Heme Oxygenase-1 Expression Protects from Type 1 Diabetes in NOD Mice
Source: Int J Mol Sci. 2019 Apr 3;20(7):1676. doi: 10.3390/ijms20071676 (PMC6480274; doi:10.3390/ijms20071676)
Supplement: Supplementary file 1 [file ijms-20-01676-s001.pdf]

International Journal of *Molecular Sciences*

Supplemental Figure 1

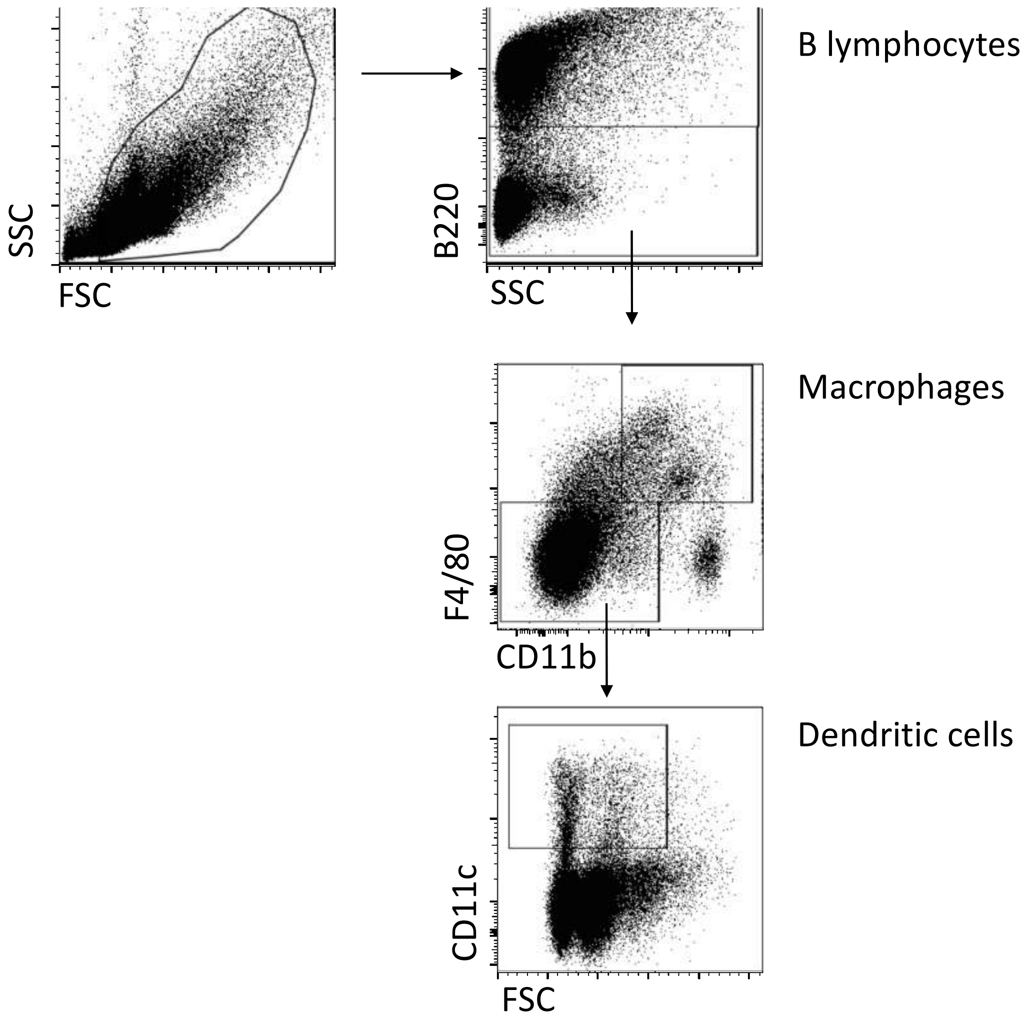

Supplemental Figure S1. Flow cytometry gating strategy used for analyzing HO-1 positive splenocytes in TetO-HO-1$^+$ pIi-tTA$^+$ NOD mice. Flow cytometry analysis of Ly6C+ cells. Splenocytes from simple TetO-HO-1$^+$ pIi-tTA$^-$ and double transgenic TetO-HO-1$^+$ pIi-tTA$^+$ NOD mice were stained with mAbs to B220, F4/80, CD11b, CD11c and analyzed by flow cytometry. Representative FACS profiles are shown.

Supplemental Figure 2

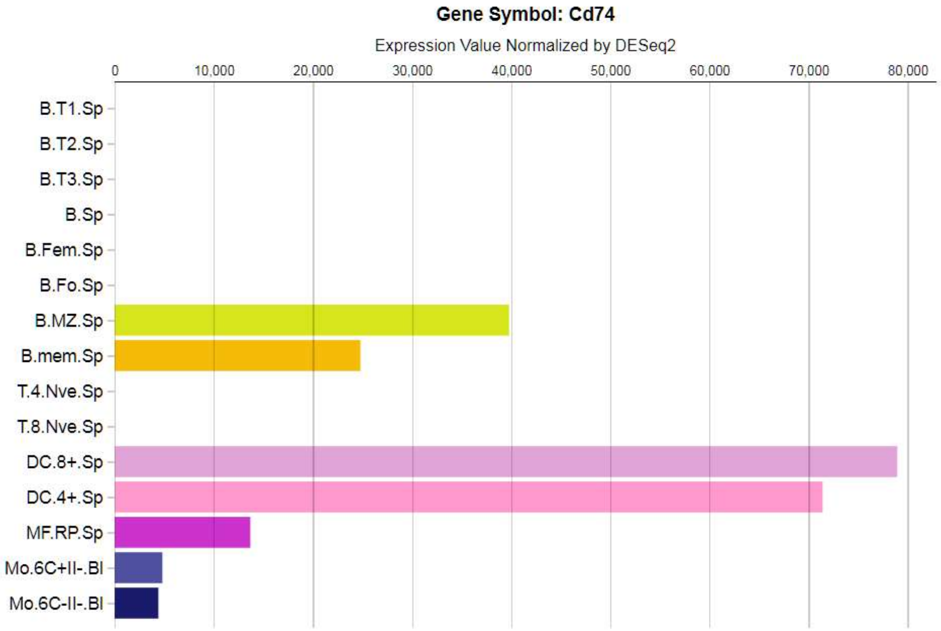

Supplemental Figure S2. Expression of MHC-II invariant chain (Eα-Ii) gene in various immune cell types. MHC-II invariant chain (Eα-Ii) (also known as CD74) mRNA abundancy in 6-weeks old C57BL/6 mice was obtained from the Immunological Genome Project (ImmGen.org). B.T1.Sp, B.T2.Sp, B.T3.Sp, B.Sp, B.Fem.Sp, B.Fo.Sp, B.MZ.Sp, B.mem.Sp were characterized in the spleen using the following surface markers: CD19+CD45R+IgM++CD93+CD23-, CD19+CD45R+IgM++CD93+CD23+, CD19+CD45R+IgM+CD93+CD23+, CD19+Igm+TCRb-, CD19+Igm+TCRb-, CD19+CD45R+IgM+CD93-CD23+CD43-CD5- and CD19+CD45R+IgM++CD93-CD23-CD21/35++ and CD19+B220+IgD-Fas-CD38+IgG+ respectively. T.4.Nve.Sp and T.8.Nve.Sp were characterized in the spleen using the following surface markers: CD4+CD8-TCRbhiCD62LhiCD44loCD25-Dump- and CD4-CD8+TCRbhiCD62LhiCD44loDump- respectively. DC.8+.Sp, DC.4+.Sp, MF.RP.Sp, were characterized in the spleen using the following surface markers: CD45+ MHCII+ CD11c+ CD8+ CD4-, CD45+ MHCII+ CD11c+ CD8- CD4+ and B220- F4/80hi MHCIIint respectively. Mo.6C+II-.Bl, Mo.6C-II-.Bl were characterized in the blood using the following surface markers: B220- CD43- CD115+ Ly-6C+ MHCII- and B220- CD43+ CD115+ Ly-6C- MHCII- respectively.
